# Association between environmental particulate matter and arterial stiffness in patients undergoing hemodialysis

**DOI:** 10.1186/s12872-015-0107-0

**Published:** 2015-10-06

**Authors:** Cheng-Hao Weng, Ching-Chih Hu, Tzung-Hai Yen, Wen-Hung Huang

**Affiliations:** Department of Nephrology, Chang Gung Memorial Hospital, Linkou, 5 Fu-Shin Street, Kwei-Shan 333, Taoyuan, Taiwan; Department of Hepatogastroenterology and Liver Research Unit, Chang Gung Memorial Hospital, Keelung, Taiwan; College of Medicine, Chang Gung University, Taoyuan, Taiwan

**Keywords:** Hemodialysis, Pulse wave velocity, Particulate matter

## Abstract

**Background:**

Aortic pulse wave velocity (PWV) has been shown to be an independent predictor of cardiovascular mortality in patients with end-stage renal disease and the general population. Atmospheric particulate- matter (PM) concentrations and their effects on cardiovascular system by affecting arterial stiffness and central hemodynamic parameters had been noted. The purpose of this study was to access the correlation of air pollution variables and PWV in patients undergoing hemodialysis (HD).

**Methods:**

This study analyzed 127 HD patients treated at the outpatient HD center. Brachial-ankle pulse wave velocity (baPWV) was measured by using a Vascular Profiler 1000 (VP-1000). Air pollution levels were recorded by a network of 27 monitoring stations near or in the patients’ living areas throughout Taiwan. The 12-month average concentrations of PM with an aerodynamic diameter of <10 and <2.5 mm (PM_10_ and PM_2.5_, respectively), sulfur dioxide (SO_2_), nitrogen dioxide (NO_2_), carbon monoxide(CO), and ozone (O_3_) were included.

**Results and Discussion:**

Multivariate linear regression analyses indicated that systolic blood pressure (SBP) (β = 0.589, P < 0.025), age (β = 0.316, *P* < 0.001), serum aluminum level (Al) (β = 0.149, *P* = 0.020), and PM_10_ (β = 0.133, *P* = 0.036) were positively correlated with baPWV.

**Conclusion:**

This cross-sectional study shows that in HD patients, the environmental PM_10_ level is associated with the baPWV.

## Background

Patients with end-stage renal disease undergoing hemodialysis (HD) have high rates of morbidity and mortality. Cardiovascular diseases account for almost half of this mortality [1]. Aortic pulse wave velocity (PWV) has been shown to be an independent predictor of cardiovascular mortality in patients with end-stage renal disease and the general population [2]. Brachial-ankle pulse wave velocity (baPWV) is an accurate indicator of aortic PWV measured by intra-aortic catheter by volume-rendering [3]. We have previous shown that serum aluminum level (Al) was positively associated with baPWV after correction of other known risk factors [4]. Adamopoulos et al. [5] analyzed the atmospheric pollution variables, including atmospheric particulate- matter (PM) concentrations and their effects on cardiovascular system by affecting arterial stiffness and central hemodynamic parameters, and found that in men, PM_10_ air pollution levels were associated with heightened amplitude of PWV. Our recently study also showed that variables of air pollution levels were associated with 2-year mortality, level of high sensitivity C-reactive protein (hsCRP), and dialysis related infections in patients undergoing peritoneal dialysis [6–8]. The purpose of this study was to access the correlation of air pollution variables and baPWV in patients undergoing HD, which had never been studied before.

## Methods

### Ethics statement

This study complied with the guidelines of the Declaration of Helsinki and was approved by the Medical Ethics Committee of Chang Gung Memorial Hospital (Institutional Review Board approval number: 101-5199B), a tertiary referral center located in the northern part of Taiwan. Written informed consent for this cross-sectional and publication of these data were obtained from every patient. All data were protected securely and only available to researchers; the data were also analyzed without patients’ names.

### Subjects

One hundred and thirty eight HD patients treated at the outpatient HD center at Chang Gung Memorial Hospital in Taoyuan, Taiwan were analyzed. To diagnose peripheral arterial occlusive disease (PAOD), the ankle-brachial blood pressure index (ABPI) was developed. PAOD has a reliable and accepted marker, which is when ABPI is less than 0.9. Severe PAOD decreases baPWV due to decreased internal pressure and blood flow. Therefore, eleven patients with ABPI less than 0.9 were excluded. The analysis enrolled 127 patients. The ESRD patients were enrolled if they were on HD for more than 3 months. Medical and demographic data were collected by chart reviews and the online database at our hospital. Regular clinical survey for all patients within one month of enrollment included serum creatinine, albumin, triglyceride and cholesterol immediately before HD. Average HD session in these patients was 4 hours and three times weekly. Our HD units use water treated by reverse osmosis. Water quality, including aluminum level less than 0.01 ppm, was proved by water analysis annually. The definition of hypotension was systolic blood pressure < 90 mmHg. The definition of intradialytic hypotension was one or more episodes of hypotension during each HD session. The definition of always hypotension was that patients had hypotension measured immediately before every HD session and throughout the entire HD session. Routine clinical workup for all patients was checked within 1 month of baPWV measurement.

### Brachial-ankle pulse wave velocity (baPWV) and ABPI measurement

Brachial-ankle pulse wave velocity and ABPI were measured by a Vascular Profiler 1000 (VP-1000) (Colin Corporation, Japan) as previously described in our study [3, 9]. Demographic data (birthday, height, weight and gender) were entered into the device. The HD patients were measured one hour before HD. After HD, baPWV does not change, or even rises. Fluid reduction by HD does not affect PWV significantly [10]. After at least 10 minutes of rest, the patients were placed in a supine position, and the value of baPWV was auto-calculated and used for analysis. This profiler records baPWV, ABPI, brachial and tibial SBP, diastolic blood pressure, pulse pressure, electrocardiogram, and phonocardiogram simultaneously. The baPWV was calculated using the equation: baPWV = (D1−D2)/t, where D1 is the distance between heart and ankle, D2 is the distance between heart and brachium, and t is the transit time between brachial arterial waves and tibial arterial waves. The ABPI was calculated as the following equation: ankle systolic pressure/arm systolic pressure. The dates of baPWV measurement were between March 1^st^, 2014 to June 30^th^, 2014. Mean arterial pressure (MAP) is widely recognized to be a determinant of arterial stiffness and we used MAP adjusted baPWV for analysis. Adjustment was performed by a linear regression of the MAP and baPWV. The residual values were then added to unadjusted baPWV to form the adjusted baPWV.

### Definition of normal and abnormal baPWV

Because there was no previous data to define the normal range of baPWV in dialysis patients, we used the reference values stated in the study by Chuang et al. [11], which showed the age and gender stratified normal reference values of baPWV derived from men and women without any of the cardiovascular risk factors for the metabolic syndrome in a community. The definition of the normal baPWV was baPWV lower than or equal to the upper limit of the reference values and the definition of abnormal baPWV was baPWV higher than the reference values.

### Air quality status and analysis

Levels of air pollution were recorded as described in our previous study [7] by a network of 26 monitoring stations near the patients’ living areas in Taiwan. Data from the database on the air quality status of Taiwan Air Quality Monitoring Network were analyzed. Due to no previous survey focused on this issue, the previous average exposure of 365 days concentration of PMs, based on the date of baPWV measurement, was used for each subject. Previous 12-month average concentrations of PM with PM with an aerodynamic diameter of <10 and <2.5 mm (PM_10_ and PM_2.5_, respectively), sulfur dioxide (SO_2_), nitrogen dioxide (NO_2_), carbon monoxide (CO), and ozone (O_3_) were included as reference items. Air pollution levels were recorded by a network of 27 monitoring stations near or in the patients’ living areas throughout Taiwan. Therefore, the average of approximately 8760 (24 × 365 = 8760) pieces of data for every monitoring station were calculated. The reference items were generally obtained from monitoring stations in the same area. If a patient lived between 2 monitoring stations, we selected the air pollutant data from the nearest station for analysis. If there is no monitoring station in a patient’s living district, we selected the reference from the nearest station (<15 km).

### Statistical analysis

Mean ± standard deviation or number and percentage in parentheses, unless otherwise stated, were used to express data. Normal distribution using the Kolmogorov–Smirnov test was tested for all variables. To compare the means of continuous variables and normally distributed data, the Student’s t test was used and the Mann–Whitney U test was used for non-normally distributed data. The chi-square test was used to analyze categorical data. Univariate linear regression analysis risk was used to assessed risk factors, and statistically significant variables (*P* < 0.05) were included in a multivariate analysis by applying a forward elimination multiple linear regression. All statistical tests were 2-tailed, with P values <0.05 being considered statistically significant. Data were analyzed using SPSS 12.0 software (SPSS, Inc., Chicago, IL).

## Results

### Subject characteristics

A total of 127 patients from a single HD center were enrolled in this study. Table 1 lists the characteristics of the study subjects (mean age, 58.5 ± 9.9 years). Of all patients, 52 were male. The median baPWV was 1767.2 ± 651.5 cm/s. The median concentration of NO_2_ was 22.9 ± 3.8 ppb; CO, 0.6 ± 0.2 ppm; SO_2_, 6.9 ± 2.1 ppb; PM_10,_ 57.9 ± 5.7 mg/m^3^; PM_2.5_, 31.8 ± 2.8 mg/m3; O_3_, 25.9 ± 2.9 ppb; and NO, 10.1 ± 7.8 ppb. Because distribution of triglyceride, intact parathyroid hormone (iPTH), Al and high sensitivity C-reactive protein (hsCRP) were skewed, they were log-transformed for further analysis.

**Table 1:** Characteristics of the studied population

Characteristic	Studied patients (n = 127)
Age (y)	58.5 ± 9.9
Male sex (%)	40.9
baPWV (cm/s)	1767.2 ± 651.5
DM (%)	23.6
Inradialytic hypotension (%)	20.5
Always hypotension (%)	3.9
HDF (%)	16.5
Hb (g/dL)	10.5 ± 1.3
BUN (mg/dL)	64.7 ± 15.8
Cr (mg/dL)	10.8 ± 2.4
Na (mEq/L)	139.8 ± 3.1
K (mEq/L)	4.8 ± 0.7
Calcium (mg/dL)	9.8 ± 1.0
Phosphorous (mg/dL)	4.6 ± 1.4
Calcium-phosphorous product (mg/dL)2	46.0 ± 16.3
Alb (g/dL)	4.1 ± 0.3
Total cholesterol (mg/dL)	185.3 ± 38.6
Triglyceride (mg/dL)	211.86 ± 157.37
Ultrafiltration amount per dialysis session (L)	2.3 ± 1.1
SBP (mmHg)	139.2 ± 27.0
ABI	1.0 ± 0.1
TBI	0.7 ± 0.1
Body Mass Index (kg/m^2^)	22.6 ± 3.5
iPTH (pg/mL)	191.3 ± 219.2
Urea reduction rate	0.8 ± 0.1
Kt/V	1.8 ± 0.3
Net protein catabolic rate (g/day/kg body weight)	1.2 ± 0.4
Aluminum (ng/mL)	10 ± 8
Hemodialysis duration (months)	72.5 ± 61.8
hsCRP (mg/L)	4.7 ± 6.4
NO_2_ (ppb)	22.9 ± 3.8
CO (ppm)	0.6 ± 0.2
SO_2_ (ppb)	6.9 ± 2.1
PM_10_ (ug/m^3^)	57.9 ± 5.7
PM2.5 (ug/m^3^)	31.8 ± 2.8
O_3_ (ppb)	25.9 ± 2.9
NO (ppb)	10.1 ± 7.8

*DM* = diabetes mellitus, *HDF* = hemodiafiltration, *Hb* = hemoglobulin, *BUN* = blood urea nitrogen, *Cr* = creatinine, *K* = potassium, *SBP* = systolic blood pressure, *Alb* = albumin, *ABI* = ankle brachial index, *TBI* = tibial brachial index, *iPTH* = intact parathyroid hormone, *Kt/V* = a number used to quantify hemodialysis treatment adequacy, *Al* = aluminum, *hsCRP* = high sensitivity C reactive protein, *NO*_*2*_ = environmental nitrogen dioxide, *CO* = environmental carbon dioxide, *SO*_*2*_ = environmental sulfur dioxide, *PM*_*10*_ = particulate matter with aerodynamic diameter <10 mm, *PM*_*2.5*_ = particulate matter with aerodynamic diameter <2.5 mm, *O*_*3*_ = environmental ozone, *NO* = environmental nitrogen oxide

We divided the patients into 2 groups, normal baPWV and abnormal baPWV. There were 59 patients in the normal baPWV group and 68 patients in the abnormal baPWV group. The levels of age, baPWV, SBP and hsCRP, and percentage of male, and DM were significantly higher in abnormal baPWV group. The percentage of intradialytic hypotension, and always hypotension were significantly higher in normal baPWV group (Table 2).

**Table 2:** Characteristics of the normal and abnormal PWV patients

Characteristic	Studied patients (n = 127)		
	Normal PWV (n = 59)	Abnormal PWV (n = 68)	*P*
Age (y)	56.4 ± 10.9	60.3 ± 8.7	0.032
Male sex (%)	30.5	48.5	0.039
baPWV (cm/s)	1247.2 ± 292.0	2218.4 ± 528.7	<0.001
DM (%)	10.2	35.3	0.001
Inradialytic hypotension (%)	34.5	8.8	<0.001
Always hypotension (%)	8.8	0	0.013
HDF (%)	18.6	14.7	0.551
Hb (g/dL)	10.5 ± 1.4	10.5 ± 1.3	0.744
BUN (mg/dL)	65.0 ± 15.7	64.5 ± 16.1	0.864
Cr (mg/dL)	11.1 ± 2.1	10.6 ± 2.7	0.223
Na (mEq/L)	139.6 ± 3.2	140.0 ± 3.1	0.518
K (mEq/L)	4.8 ± 0.7	4.9 ± 0.6	0.511
Calcium (mg/dL)	9.7 ± 1.0	9.9 ± 1.0	0.276
Phosphorous (mg/dL)	4.6 ± 1.5	4.6 ± 1.4	0.815
Calcium-phosphorous product (mg/dL)2	46.7 ± 16.5	45.4 ± 16.2	0.667
Alb (g/dL)	4.0 ± 0.2	4.1 ± 0.4	0.429
Total cholesterol (mg/dL)	180.5 ± 39.1	189.4 ± 38.0	0.199
Triglyceride (mg/dL)	195.5 ± 130.0	226.1 ± 177.5	0.276
Ultrafiltration amount per dialysis session (L)	2.5 ± 1.2	2.0 ± 0.9	0.017
SBP (mmHg)	129.2 ± 29.3	147.9 ± 21.5	<0.001
ABI	1.00 ± 0.15	1.05 ± 0.12	0.055
TBI	0.72 ± 0.16	0.73 ± 0.15	0.545
Body Mass Index (kg/m^2^)	22.6 ± 3.1	22.5 ± 3.4	0.916
iPTH (pg/mL)	188.6 ± 201.9	193.7 ± 234.6	0.898
Urea reduction rate	0.78 ± 0.06	0.78 ± 0.06	0.747
Kt/V	1.8 ± 0.3	1.8 ± 0.4	0.655
Net protein catabolic rate (g/day/kg body weight)	1.2 ± 0.4	1.2 ± 0.4	0.946
Al (ng/mL)	8.8 ± 5.7	10.4 ± 8.9	0.232
Hemodialysis duration (months)	78.7 ± 67.8	67.2 ± 56.0	0.298
hsCRP (mg/L)	3.4 ± 3.5	5.8 ± 7.7	0.023
NO_2_ (ppb)	22.9 ± 3.6	22.9 ± 4.0	0.984
CO (ppm)	0.63 ± 0.17	0.64 ± 0.22	0.890
SO_2_ (ppb)	6.7 ± 2.0	7.2 ± 2.1	0.184
PM_10_ (ug/m^3^)	57.2 ± 5.4	58. ± 5.9	0.226
PM_2.5_ (ug/m^3^)	31.6 ± 2.9	31.9 ± 2.8	0.513
O_3_ (ppb)	25.7 ± 2.8	26.0 ± 2.9	0.480
NO (ppb)	9.9 ± 4.7	10.4 ± 9.8	0.707

*Normal PWV* = within the distribution of PWV, *Abnormal PWV*: higher than the distribution of PWV, *DM* = diabetes mellitus, *HDF* = hemodiafiltration, *Hb* = hemoglobulin, *BUN* = blood urea nitrogen, *Cr* = creatinine, *K* = potassium, *SBP* = systolic blood pressure, *Alb* = albumin, *ABI* = ankle brachial index, *TBI* = tibial brachial index, *iPTH* = intact parathyroid hormone, *Kt/V* = a number used to quantify hemodialysis treatment adequacy, *Al* = aluminum, *hsCRP* = high sensitivity C reactive protein, *NO*_*2*_ = environmental nitrogen dioxide, *CO* = environmental carbon dioxide, *SO*_*2*_ = environmental sulfur dioxide, *PM*_*10*_ = particulate matter with aerodynamic diameter <10 mm, *PM*_*2.5*_ = particulate matter with aerodynamic diameter <2.5 mm, *O*_*3*_ = environmental ozone, *NO* = environmental nitrogen oxide

### Factors associated with baPWV level in patients undergoing HD

Univariate linear regression identified several clinical variables that were significantly associated with baPWV. These included fasting glucose (β = 0.272, *P* = 0.002), gender (β = 0.250, female as reference, *P* = 0.005), age (β = 0.375, *P* < 0.001), log-transformed Al (log Al) (β = 0.201, *P* = 0.023), diabetes mellitus (β = 0.293, *P* = 0.001), intradialytic hypotension (β = −0.209, *P* = 0.019), log transformed hsCRP (log hsCRP) (β = 0.229, *P* = 0.010), SO_2_ (β = 0.208, *P* = 0.019), heart rate (β = 0.545, *P* = <0.001) and PM_10_ (β = 0.205, *P* = 0.021). Multivariate linear regression analyses indicated that heart rate (β = 0.433, *P* < 0.001), intradialytic hypotension (β = −0.198, *P* = 0.006), age (β = 0.193, *P* = 0.013), log Al (β = 0.144, *P* = 0.045), and PM_10_ (β = 0.150, *P* = 0.035) were positively correlated with baPWV (Table 3).

**Table 3:** Linear regression analysis with baPWV as the dependent variable

Variable	Unstandardized coefficients		Standardized coefficients	*P* value
	B	Std. error	Beta	
Univariate				
Fasting glucose (mg/dL)	2.758	0.873	0.272	0.002
Gender (female as reference group)	330.763	114.639	0.250	0.005
Age (y)	24.567	5.426	0.375	<0.001
Log Al (ng/mL)	417.573	181.613	0.201	0.023
DM	447.395	130.649	0.293	0.001
Intra-dialytic hypotension	−334.493	140.764	−0.209	0.019
Log hsCRP (mg/L)	308.112	116.982	0.229	0.010
SO_2_ (ppb)	65.722	27.617	0.208	0.019
PM_10_ (ug/m^3^)	23.512	10.043	0.205	0.021
Heart rate (/min)	33.727	4.647	0.545	<0.001
Log triglyceride (mg/dL)	60.834	199.684	0.027	0.761
Hb (g/dL)	12.615	44.066	0.026	0.775
Alb (g/dL)	−76.401	179.385	−0.038	0.671
Total cholesterol (mg/dL)	−0.373	1.510	−0.022	0.805
Calcium (mg/dL)	27.205	59.121	0.041	0.646
Phosphorous (mg/dL)	−56.563	40.098	−0.125	0.161
Calcium-phosphorous product (mg/dL)2	−5.512	3.573	−0.137	0.125
Body Mass Index (kg/m^2^)	22.999	16.460	0.124	0.165
Urea reduction rate	−1100.164	999.288	−0.098	0.273
Kt/V	−244.671	172.193	−0.127	0.158
Net protein catabolic rate (g/day/kg body weight)	−197.253	156.598	−0.116	0.210
Hemodialysis duration (months)	−1.334	0.936	−0.126	0.156
NO_2_ (ppb)	−5.595	15.351	−0.033	0.716
CO (ppm)	−163.429	295.340	−0.050	0.581
PM_2.5_ (ug/m^3^)	22.878	20.493	0.099	0.266
O_3_ (ppb)	23.474	20.080	0.104	0.245
NO (ppb)	−1.728	7.433	−0.021	0.817
ARB/ACEi	246.279	173.512	0.216	0.158
CCB	−7.177	145.965	−0.004	0.961
Beta blocker	47.806	139.934	0.031	0.733
Multivariate				
Heart rate (/min)	26.849	4.775	0.433	<0.001
Intra-dialytic hypotension	−317.174	112.815	−0.198	0.006
Age (y)	12.615	4.978	0.193	0.013
Log Al (ng/mL)	297.553	146.640	0.144	0.045
PM_10_ (ug/m^3^)	17.517	8.234	0.150	0.035

*DM* = diabetes mellitus, *SBP* = systolic blood pressure, *log Al* = log-transformed aluminum, log *hsCRP* = log-transformed high sensitivity C reactive protein, *Log triglyceride* = log-transformed triglyceride, *SO2* = environmental sulfur dioxide, *PM*_*10*_ = particulate matter with aerodynamic diameter <10 mm, *O*_*3*_ = environmental ozone, *Hb* = hemoglobulin, *Alb* = albumin, *NO*_*2*_ = environmental nitrogen dioxide, *CO* = environmental carbon dioxide, *PM*_*2.5*_ = particulate matter with aerodynamic diameter <2.5 mm, *NO* = environmental nitrogen oxide, *Kt/V* = a number used to quantify hemodialysis treatment adequacy, *ARB* = angiotensin receptor blocker, *ACEi* = angiotensin-converting-enzyme inhibitor, *CCB* = calcium channel blocker

## Discussion

The purpose of the present study was to assess the cross sectional relations between clinical variables, ambient PM_10_ concentrations, and baPWV in HD patients. The main findings of the present study were that: PM_10_, age, Al and SBP were independently correlated with baPWV and higher concentrations of ambient PM_10_ was associated with a higher magnitude of baPWV.

This study is the first to show that environmental PM_10_ is positively associated with baPWV in HD patients. Particulate matter inhalation has been associated with acute arterial vasoconstriction in healthy adults [12], disrupting systolic function [13], heart rate variability [14], and persistent lung inflammation and endothelial dysfunction [15], factors that may increase the PWV. Automobile emissions are the most important source of PM_10_ in the urban areas, followed by crustal materials, secondary aerosols, biomass burning, industrial emissions and marine spray in Taiwan [16]. Lanqrishet et al. [17] showed that following exposure to diesel exhaust, N(G)-monomethyl-l-arginine (l-NMMA), a NO synthase inhibitor, caused increase in blood pressure and arterial stiffness. Graff *et a*l. [18] demonstrated that after 2-hours of exposure to crustal materials, mild pulmonary inflammation, decreased tissue plasminogen activator, and decreased heart rate variability. Heo et al. [19] showed that particles derived from mobile sources (i.e., gasoline and diesel emissions) and biomass burning were associated with respiratory mortality and cardiovascular mortality, respectively. The cardiovascular mortality may be due to the increased PWV as observed in our study. Ambient PM_10_ exposure had also been reported to induce considerable oxidative stress and systemic inflammation in ApoE knockout mice and contributed to the progression of atherosclerosis [20]. Systemic inflammation and atherosclerosis are both predictors of increased PWV [21]. Adamopoulos et al. showed no significant association between environmental variables and arterial stiffness. However, in men, the mean 5- day PM_10_ air concentration was independently associated with the augmentation pressure [2.0 mmHg (95 % confidence interval (CI) 0.56–3.39) per 43.4 mg/m^3^] and the aortic-pulse pressure [2.78 mmHg (95 % CI 3.91–5.12)] denoting a significant effect of PM on the aortic-wave reflection magnitude and central hemodynamics [5]. In our study, we have demonstrated that PM_10_ was associated with baPWV, including men and women undergoing HD. The difference between our study and Adamopoulos’s might be the more susceptible to the influence by air pollution in HD patients.

In our previous study, we showed that living in Taipei Basin was a risk factor predicting 2-year mortality in elderly HD patients [22]. Air pollution in this crowded area may be the factor that caused this phenomenon. The present study also showed that age was also significantly correlated with baPWV. Therefore, higher PWV caused by PM_10_ might be a reason for higher 2-year mortality in HD patients living in Taipei Basin area. Our studies also demonstrated that environmental NO_2_ level was associated with 2-year mortality [8] and environmental CO level was associated with the level of hsCRP in peritoneal dialysis patients [6].

This study showed that Al was positively associated with baPWV and the correlation between Al and baPWV had been discussed in our previous study [4]. In the study by Michael et al. [23], aluminum was one of the components of PM_10_. Therefore, we calculated the correlation between serum Al level and PM_10_ and showed no significant correlation. The serum aluminum of these patients did not come from air pollution and might be due to medication, drinking water, or dissociation from aluminum containers.

## Conclusion

In conclusion, this cross-sectional study showed that in HD patients, the environmental PM_10_ level was associated with baPWV.

## Abbreviations

PWV: Aortic pulse wave velocity
PM: Particulate- matter
HD: Hemodialysis
baPWV: Brachial-ankle pulse wave velocity
PM_10_ and PM_2.5_: An aerodynamic PM diameter of <10 and <2.5 mm
SO_2_: Sulfur dioxide
NO_2_: Nitrogen dioxide
CO: Carbon monoxide
O_3_: Ozone
SBP: Systolic blood pressure
Al: Serum aluminum level
hsCRP: High sensitivity C-reactive protein
ABPI: Ankle-brachial blood pressure index
PAOD: Peripheral arterial occlusive disease
